# Isolation and identification of two pairs of cytotoxic diterpene tautomers and their tautomerization mechanisms

**DOI:** 10.1038/s41598-020-58260-8

**Published:** 2020-01-29

**Authors:** Li-Ping Dai, Xiao-Fei Li, Qing-Mei Feng, Ling-Xia Zhang, Qiu-Yan Liu, Er-Ping Xu, Hong Wu, Zhi-Min Wang

**Affiliations:** 1 School of Pharmacy, Henan University of Traditional Chinese Medicine, Zhengzhou, 450046 China; 2 Research Center for Classic Chinese Medicines & Health Herbal Products, Henan University of Traditional Chinese Medicine, Zhengzhou, 450046 China; 3 Institute of Chinese Materia Medica, China Academy of Chinese Medical Sciences, Beijing, 100700 China; 4 The Third Affiliated Hospital of Henan University of Traditional Chinese Medicine, Henan, China

**Keywords:** Target identification, Secondary metabolism

## Abstract

Discovering anticancer drugs that do not have adverse side effects has been a developing research field worldwide in recent decades. In this work, four previously undescribed cytotoxic diterpenoids were isolated from the aerial parts of *Isodon excisoides*. Interestingly, these four diterpenoids were two pairs of tautomers that were first reported in plants. Their structures were further elucidated using various spectroscopic methods. The tautomerization phenomenon and mechanism for these two pairs of tautomers were emphatically described. The theoretical simulation results indicated that the diterpene tautomerization is greatly related to certain factors, including the existence of a transition state, the change of bond length and the level of conversion energy; the tautomerization for the two pairs of tautomers is mainly caused by proton transfer. For bioassays, the cytotoxicities of the tautomers against five human cancer cell lines were also investigated. The results indicated that each of the four diterpenoids showed significant cytotoxicity in at least three cell lines and could serve as potential anticancer agents for further investigation.

## Introduction

Globally, cancer is the leading cause of morbidity and mortality. For certain types of cancer, chemotherapy drugs have been extensively used for treatment. However, resistance to chemotherapy and severe side effects are the drawbacks of these agents. Therefore, the development of new agents derived from plants has intensified.

Natural and synthetic tetracyclic diterpenoids exhibit interesting pharmacological activities^1^. The plants of the *Isodon* genus are rich in diterpenoids. In addition, ent-kaurane diterpenoids, containing an enone system in ring D, are a class of characteristic components found in this genus, and they are mainly responsible for the reported cytotoxic activity^2^. To date, approximately 1000 diterpenoids from the *Isodon* genus have been shown to exhibit significant cytotoxicity^2–8^, and many diterpenoids with anticancer activities, such as oridonin, erycalyxin A and rabdophyllin G, have also been developed into new drugs^9,10^. *Isodon excisoides*, a common wild species, is a perennial herb mainly distributed in the western region of the Henan and Yunnan Provinces in China. Local inhabitants in the western mountainous region of Henan Province generally believe that *I. excisoides* is better for preventing esophageal cancer than that of *Isodon rubescens* from the same genus. Our previous studies showed that many diterpenoids from *I. excisoides* have anticancer activities^2^. As part of our ongoing search for anticancer diterpenoids, four new diterpenes, as two pairs of tautomers, were purified and identified from *I. excisoides* in this work. Moreover, the tautomerization phenomenon for both pairs of tautomers was also observed. It is worth mentioning that the tautomerism of diterpenoids was observed for the first time. Simultaneously, the tautomerization mechanism was emphatically discussed. Finally, their cytotoxicities against five human cancer cell lines were also investigated.

## Results and Discussion

### Structural elucidation of new compounds

**Figure 1:**
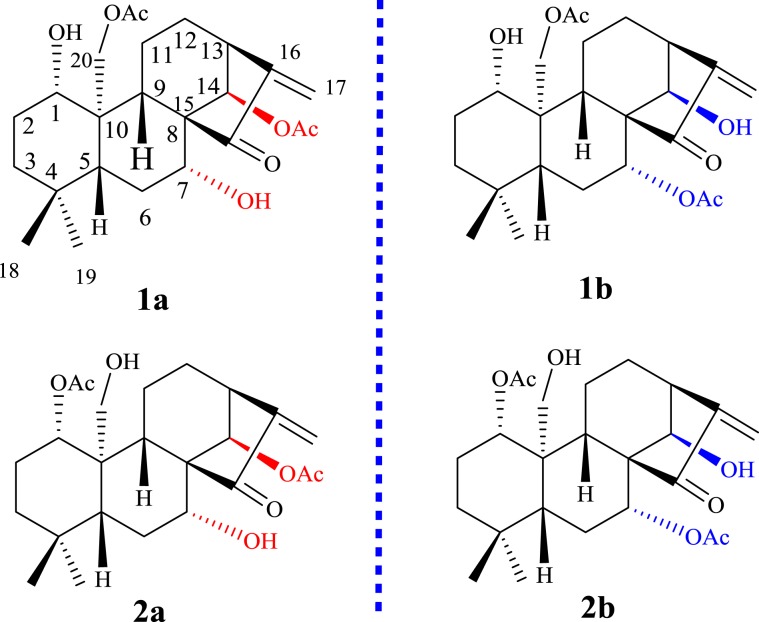
Structures for two pairs of tautomers.

In this paper, four undescribed 7,20-non-epoxy-*ent*-kaurane skeleton tautomeric diterpenes [**1a** (45 mg) and **1b** (30 mg) and **2a** (42 mg) and **2b** (31 mg)] (Fig. 1) were isolated from the aerial parts of *I. excisoides* utilizing various chromatographic methods, including D-101 macroporous adsorptive resins, silica gel, sephadex LH-20 and semi-preparative HPLC. The structures of **1a**, **1b**, **2a** and **2b** were elucidated by nuclear magnetic resonance spectroscopy and high-resolution mass spectrometry in conjunction with published data of their analogues, as well as their fragmentation patterns. As the result, the four compounds were elucidated to be 1*α*,7*α*-dihydroxy-14*β*,20-diacetoxy-*ent*-kaur-15-one (**1a**), 1*α*,14*β*-dihydroxy-7*α*,20-diacetoxy-*ent*-kaur-15-one (**1b**), 1*α*,14*β*-diacetoxy-7*α*,20-dihydroxy-*ent*-kaur-16-en-15-one (**2a**), and 1*α*,7*α*-diacetoxy-14*β*,20-dihydroxy-*ent*-kaur-16-en-15-one (**2b**) (Fig. 1, Tables 1 and 2).

**Table 1:** NMR spectroscopic data for compound **1a** and **1b**.

No.	1a		No.	1b	
	*δ* C^a,b^	*δ* H^a,c^ (*J* in Hz)		*δ* C^a,b^	*δ* H^a,c^ (*J* in Hz)
1	81.5 (d)	3.35 (1H, dd, 5.5, 9.5)	1	81.5 (d)	3.36 (1H, dd, 5.4, 10.9)
2	30.5 (t)	1.88 (1H, overlapped) 1.62 (1H, m)	2	30.8 (t)	1.99 (1H, overlapped) 1.72 (1H, m)
3	39.3 (t)	1.49 (1H, overlapped) 1.28 (1H, dt, 4.3, 9.5)	3	39.1 (t)	1.46 (1H, overlapped) 1.32 (1H, dt, 4.1, 9.6)
4	33.0 (s)	−	4	33.0 (s)	−
5	52.4 (d)	0.98 (1H, dd, 1.7, 10.7)	5	51.7 (d)	1.11 (1H, dd, 2.0, 12.0)
6	28.3 (t)	2.00 (1H, q, 12.5) 1.88 (1H, overlapped)	6	25.4 (t)	1.99 (2H, overlapped)
7	73.7 (d)	5.12 (1H, m)	7	76.4 (d)	5.37 (1H, dd, 4.5, 11.8)
8	61.5 (s)	−	8	61.0 (s)	−
9	56.4 (d)	1.83 (1H, d, 8.4)	9	55.5 (d)	1.77 (1H, d, 8.5)
10	45.8 (s)	−	10	45.8 (s)	−
11	19.6 (t)	2.89 (1H, dd, 5.2, 10.8) 1.49(1H, overlapped)	11	19.5 (t)	2.86 (1H, dd, 5.1, 11.9) 1.46 (1H, overlapped)
12	31.8 (t)	2.13 (1H, m) 1.73 (1H, m)	12	31.8 (t)	1.84 (1H, dt, 3.8, 12.3) 1.66 (1H, m)
13	44.1 (d)	3.09 (1H, br.s)	13	44.1 (d)	3.09 (1H, br.s)
14	78.2 (d)	6.00 (1H, br.s)	14	75.0 (d)	4.80 (1H, br.s)
15	207.0 (s)	−	15	205.9 (s)	−
16	146.3 (s)	−	16	146.9 (s)	−
17	117.9 (t)	6.15 (1H, s) 5.38 (1H, s)	17	116.5 (t)	6.14 (1H, s) 5.42 (1H, s)
18	33.3 (q)	0.88 (3H, s)	18	33.0 (q)	0.91 (3H, s)
19	21.3 (q)	0.81 (3H, s)	19	21.3 (q)	0.81 (3H, s)
20	63.9 (t)	4.60 (1H, d, 13.6) 4.54 (1H, d, 13.6)	20	64.1 (t)	4.57 (1H, d, 13.4) 4.43 (1H, d, 13.4)
20-OAc	171.3 (s)	−	20-OAc	170.7 (s)	−
20-OAc	21.5 (q)	2.16 (3H, s)	20-OAc	21.6 (q)	2.17 (3H, s)
14-OAc	170.2 (s)	−	7-OAc	168.3 (s)	−
14-OAc	21.6 (q)	1.99 (3H, s)	7-OAc	21.3 (q)	2.00 (3H, s)
7-OH	−	2.45 (1H, d, 6.6)	14-OH	−	3.97 (1H, s)
1-OH	−	1.39 (1H, d, 5.5)	1-OH	−	1.41 (1H, d, 5.4)

^a^Measured in CDCl3.

^b^Measured at 125 MHz.

^c^Measured at 500 MHz.

**Table 2:** NMR spectroscopic data for compound **2a** and **2b**.

No.	2a		No.	2b	
	*δ* C^a,b^	*δ* H^a,c^ (*J* in Hz)		*δ* C^a,b^	*δ* H^a,c^ (*J* in Hz)
1	84.1 (d)	4.62 (1H, dd, 5.8, 9.4)	1	83.8 (d)	4.61 (1H, dd, 5.0, 10.0)
2	25.4 (t)	1.77 (2H, overlapped)	2	25.7 (t)	1.76 (2H, overlapped)
3	38.3 (t)	1.47 (1H, overlapped) 1.32 (1H, td, 5.6, 12.6)	3	38.2 (t)	1.46 (1H, overlapped) 1.35 (1H, td, 4.8, 12.8)
4	33.0 (s)	−	4	32.9 (s)	−
5	52.8 (d)	1.04 (1H, d, 10.6)	5	52.7 (d)	1.17 (1H, d, 11.5)
6	27.5 (t)	1.89 (1H, d, 12.6) 1.83 (1H, m)	6	25.3 (t)	2.01 (1H, m) 1.76 (1H, overlapped)
7	73.2 (d)	4.21 (1H, d, 12.5)	7	76.4 (d)	5.41 (1H, dd, 4.5, 11.8)
8	61.5 (s)	−	8	60.9 (s)	−
9	55.9 (d)	1.78 (1H, d, 8.2)	9	55.2 (d)	1.76 (1H, overlapped)
10	45.8 (s)	−	10	47.2 (s)	
11	20.5 (t)	1.70 (1H, dd, 4.7, 15.2) 1.44 (1H, overlapped)	11	20.2 (t)	1.46 (1H, overlapped)
12	30.6 (t)	2.71 (1H, 1H, tt, 5.5, 7.3) 1.61 (1H, m)	12	30.4 (t)	2.54 (1H, overlapped) 1.56 (1H, m)
13	38.3 (d)	3.10 (1H, br.s)	13	38.2 (d)	3.11 (1H, br.s)
14	76.9 (d)	6.02 (1H, br.s)	14	75.0 (d)	4.99 (1H, br.s)
15	205.0 (s)	−	15	205.8 (s)	−
16	146.3 (s)	−	16	147.1 (s)	−
17	117.4 (t)	6.11 (1H, s) 5.39 (1H, s)	17	118.0 (t)	6.12 (1H, s) 5.41(1H, s)
18	33.3 (q)	0.88 (3H, s)	18	33.0 (q)	0.92 (3H, s)
19	21.9 (q)	0.86 (3H, s)	19	22.0 (q)	0.85 (3H, s)
20	61.1 (t)	4.44 (1H, d, 13.6) 4.27 (1H, dd, 2.4, 13.6)	20	61.6 (t)	4.00 (1H, d, 13.6) 4.39 (1H, dd, 2.4, 13.6)
1-OAc	171.3 (s)	−	1-OAc	168.9 (s)	−
1-OAc	22.1 (q)	2.03 (3H, s)	1-OAc	22.0 (q)	2.02 (3H, s)
14-OAc	170.4 (s)	−	7-OAc	168.2 (s)	−
14-OAc	21.6 (q)	1.96 (3H, s)	7-OAc	21.8 (q)	1.99 (3H, s)

^a^Measured in CDCl3.

^b^Measured at 125 MHz.

^c^Measured at 500 MHz.

Compound **1a** was a white powder. The molecular formula of **1a** was determined to be C_24_H_34_O_7_ on the basis of positive HRESIMS at *m/z* 457.21759 [M + Na]^+^ (calcd for C_24_H_34_O_7_Na^+^, *m/z* 457.21967). The UV spectrum of **1a** showed an absorption maximum at 235 nm. The IR spectrum of **1a** showed the presence of hydroxyl (3445 cm^−1^), carbonyl (1729 cm^−1^) and double bond (1649 cm^−1^) groups. The ^1^H, ^13^C-NMR and HSQC spectra of **1a**, together with the results from an HMBC experiment showed the presence of one exocyclic double bond [*δ*_H_ 6.15 (1H, brs), 5.38 (1H, brs); *δ*_C_ 117.9, 146.3], two angular methyl groups [*δ*_H_ 0.88 (3H, s) and 0.81 (3H, s); *δ*_C_ 33.3 (q) and 21.3 (q)], one ketone carbonyl (*δ*_C_ 207.0) and two acetoxyl groups [*δ*_H_ 2.16 (3H, s); *δ*_C_ 171.3 (s), 21.5 (q); *δ*_H_ 1.99 (3H, s); *δ*_C_ 170.2 (s), 21.6 (q)]. In addition, the other carbon signals were assigned to six methenes including three oxy-methines (*δ*_C_ 81.7, 73.7, 78.2) and six methylene carbons including one oxy-methylene (*δ*_C_ 63.9), and three quaternary carbons (*δ*_C_ 61.7, 46.1, 33.1). Considering the diterpenoids previously isolated from the plant, **1a** was tentatively presumed to be a 7,20-non-epoxy-*ent*-kaurane skeleton, substituted with two hydroxyl groups and two acetoxyl groups.

**Figure 2:**
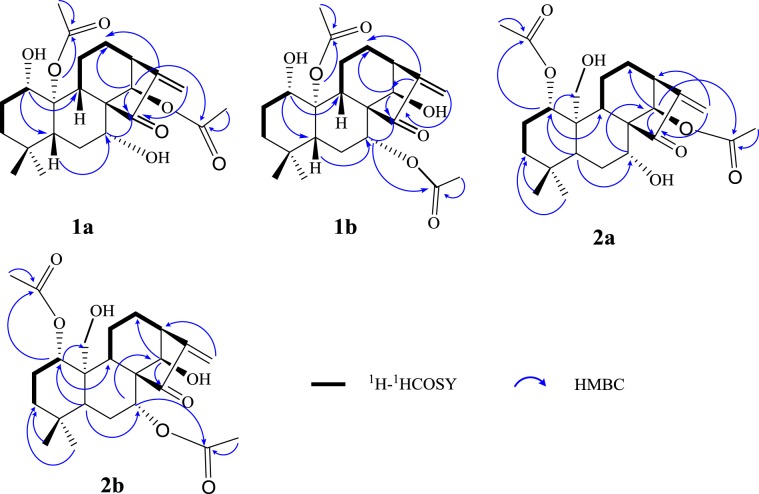
Key ^1^H-^1^H COSY and HMBC for two pairs of tautomers.

The ^1^H-NMR and ^13^C-NMR data of **1a** were nearly identical to that of henryin, a known diterpene^11^, and the only difference found was in the moiety at C-14. The compounds **1a** was a 14-acetylated compound of henryin. This hypothesis was further confirmed by the HMBC spectra of **1a**. In the HMBC spectrum (Fig. 2), the correlations for *δ*_H_ 5.99 (H-14) with *δ*_C_ 146.3 (C-16), *δ*_C_ 207.0 (C-15) and *δ*_C_ 170.2 (-OO**C**CH_3_) revealed that the hydroxyl group at C-14 in henryin [*δ*_C_ 75.8; 4.78 (1H, s)] had been replaced by an acetoxyl group in **1a** [*δ*_H_ 1.99 (3H, s); *δ*_C_ 170.2 (s), 21.6 (q)]. Thus, the basic skeleton of **1a** was assumed to be 1,7-dihydroxy-14,20-diacetoxy-*ent*- kaur-16-en-15-one.

**Figure 3:**
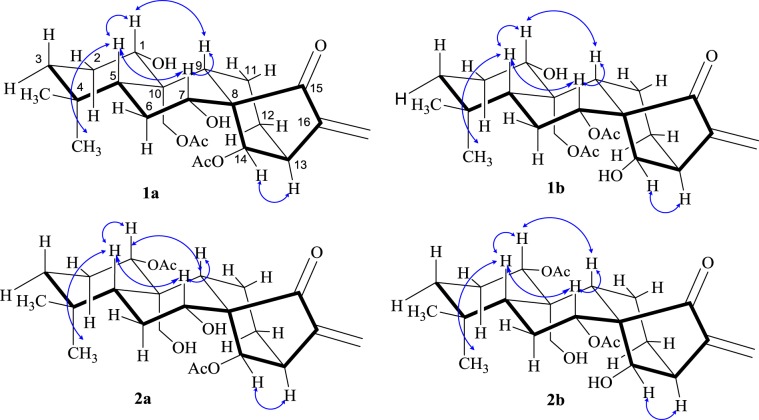
The key NOESY correlations for two pairs of tautomers.

The relative configuration of the substituents was highlighted in a NOESY spectrum. The correlations of H-1 with H-5 and H-9, Me-18 with H-5, H-7 with H-5 and H-9, and H-13 with H-14 and H-16 indicated that H-1, H-5, H-7 and H-9 were positioned on the same side and that H-13, H-14 and H-16 were on the other side (Fig. 3).

**Figure 4:**
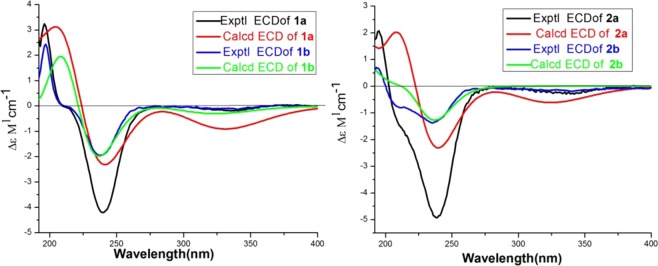
Calculated and experimental CD spectra for compounds **1a**, **1b**, **2a** and **2b** in CHCl_3_.

To determine the absolute configuration, the electronic circular dichroism (ECD) spectrum of compound **1a** was measured in MeOH and compared with the computed ECD spectra of **1a**. The calculated curve matched well with that of the experimental curve (Fig. 4). According to the octant rule for saturated cyclopentanone^4^, the negative Cotton effect at 247.94 nm, based on the n-π* transition of the saturated cyclopentanone moiety, indicated that the D ring was β-oriented (Fig. 4). Finally, the structure of compound **1a** was elucidated as 1α,7α-dihydroxy-14β,20-diacetoxy-*ent*-kaur-16-en- 15-one (Fig. 1).

Compound **1b** was a white powder. The molecular formula of **1b** was determined to be C_24_H_34_O_7_ by positive HRESIMS (*m/z* 457.21774 [M + Na]^+^, calcd for C_24_H_34_O_7_Na^+^, *m/z* 457.21967). The UV, IR, ^1^H, ^13^C-NMR, HSQC and HR-ESI-MS spectra of **1b**, together with the results from an HMBC experiment, showed that **1b** was an isomer of **1a**. Comparing the NMR data of **1a** and **1b** (Table 1). It can be found that the substituents on C-7 and C-14 were exchanged in **1a** and in **1b**. An acetoxyl group and a hydroxyl group were at C-7 and C-14 in **1b**, respectively. It was also found that in the HMBC spectrum (Fig. 2), there were correlations between *δ*_H_ 5.38 (H-7) and *δ*_C_ 51.8 (C-5), *δ*_C_ 45.6 (C-10) and *δ*_C_ 168.2 (7-CH_3_COO-), and *δ*_H_ 4.79 (H-14) and *δ*_C_ 55.7 (C-9), *δ*_C_ 205.7 (C-15) and *δ*_C_ 146.1 (C-16). Thus, the planar structure of **1b** was assumed to be 1,14-dihydroxy-7,20-diacetoxy-*ent*-kaur-16-en-15-one.

The same relative stereo-structure for **1a** and **1b** was deduced from their similar NOESY correlations (Fig. 3) and their almost identical ^1^H- and ^13^C-NMR data. In addition, compound **1b** exhibited almost the same CD absorption as that of **1a**. The calculated curve was in good agreement with that of the experimental curve (Fig. 4). Thus, the structure of 1a was determined to be 1α,14*β*-dihydroxy-7α,20-diacetoxy-*ent*-kaur-16-en-15-one (Fig. 1).

Compound **2a** was obtained as a white powder, and its molecular formula was determined to be C_24_H_34_O_7_ by positive HRESIMS (*m/z* 457.21756 [M + Na], calcd C_24_H_34_O_7_Na^+^, *m/z* 457.21967). The UV spectrum of **2a** showed an absorption maximum at 235 nm. The IR spectrum of **2a** showed the presence of hydroxyl (3445 cm^−1^), carbonyl (1730 cm^−1^) and double bond (1648 cm^−1^) groups. Together with the NMR data of **2a**, the results showed **2a** was also an analogue of **1a**.

Comparing the NMR data of compounds **1a** and **2a** (Table 1), it was found that the substituents on C-1 and C-20 were exchanged for **1a** and **2a**, respectively. The acetoxyl and hydroxyl groups were at C-1 and C-20 in **2a**, respectively. In the HMBC spectrum (Fig. 2), *δ*_H_ 4.62 (H-1) correlated with *δ*_C_ 25.4 (C-2), *δ*_C_ 47.3 (C-10), *δ*_C_ 55.9 (C-9) and *δ*_C_ 169.1 (1-CH_3_**C**OO-); *δ*_H_ 4.44 (H-20*β*) correlated with *δ*_C_ 55.9 (C-9), and *δ*_H_ 4.23 (H-20α) correlated with *δ*_C_ 84.1(C-1). Thus, the planar structure of **2a** was assumed to be 1,14-diacetoxy-7,20-dihydroxy-*ent*-kaur-16-en-15-one.

The relative configuration of the substituents of **2a** was determined with the NOESY spectrum. The correlations of H-1 with H-5 and H-9, Me-18 with H-5, H-7 with H-5 and H-9, and H-13 with H-14 and H-16 indicated that they were positioned on the same side and that H-14, H-13, and H-16 were on the other side (Fig. 3).

In addition, compound **2a** exhibited almost the same CD absorption as that of **1a**. The calculated curve matched well with that of the experimental curve (Fig. 4). Thus, the structure of **2a** was determined to be 1α,14*β*-diacetoxy-7α,20-dihydroxy-*ent*-kaur-16-en-15-one (Fig. 1).

Compound **2b** was a white powder, and its molecular formula was determined to be C_24_H_34_O_7_ by positive HRESIMS (*m/z* 457.21799 [M + Na]^+^, calcd for C_24_H_34_O_7_ Na^+^, *m/z* 457.21967). The UV, IR and HR-ESI-MS spectra, together with the NMR data of **2b**, showed that **2b** was an isomer of **2a**.

Comparing the NMR data of compounds **2a** and **2b** (Table 1), it was found that the substituents on C-7 and C-14 were exchanged with each other. An acetoxyl group and a hydroxyl group were at C-1 and C-20 in **2a**, respectively. In the HMBC spectrum (Fig. 2), *δ*_H_ 4.62 (H-1) correlated with *δ*_C_ 25.4 (C-2), *δ*_C_ 47.3 (C-10), *δ*_C_ 55.9 (C-9) and *δ*_C_ 169.1 (1-CH_3_**C**OO-); *δ*_H_ 4.44 (H-20*β*) correlated with *δ*_C_ 55.9 (C-9); *δ*_H_ 4.23 (H-20α) correlated with *δ*_C_ 84.1 (C-1). The NOESY and CD experiments showed that the configuration of the substituents of **2b** is the same as that of **2a** (Figs. 3 and 4). Thus, the structure of **2b** was assumed to be 1α,7α-diacetoxy-14*β*,20-dihydroxy-*ent*-kaur-16-en-15-one (Fig. 1).

### Tautomeric phenomenon and the dynamic equilibrium of two pairs of diterpene tautomers

**Figure 5:**
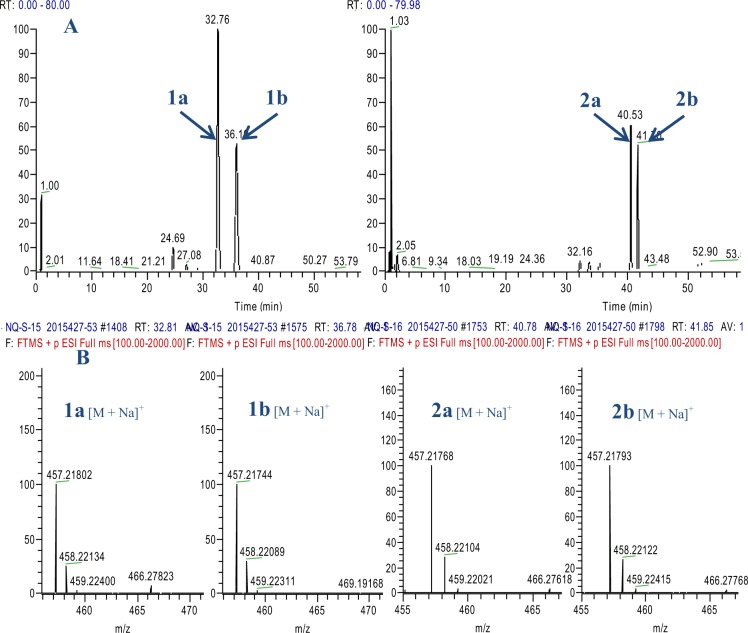
(**A**) Total ion chromatogram (TIC) of two pairs tautomers in positive ion mode using UPLC-LTQ-Orbitrap-MS; (**B**) HR-ESI-MS spectrum of two pairs tautomers.

The UPLC-MS/MS and NMR data (Fig. 5) (Figs. S1–4, Supporting information) showed that **1a** and **1b** and **2a** and **2b** were two pairs of tautomers. The interconversion phenomenon between each pair of diterpene tautomers was observed by HPLC.

**Figure 6:**
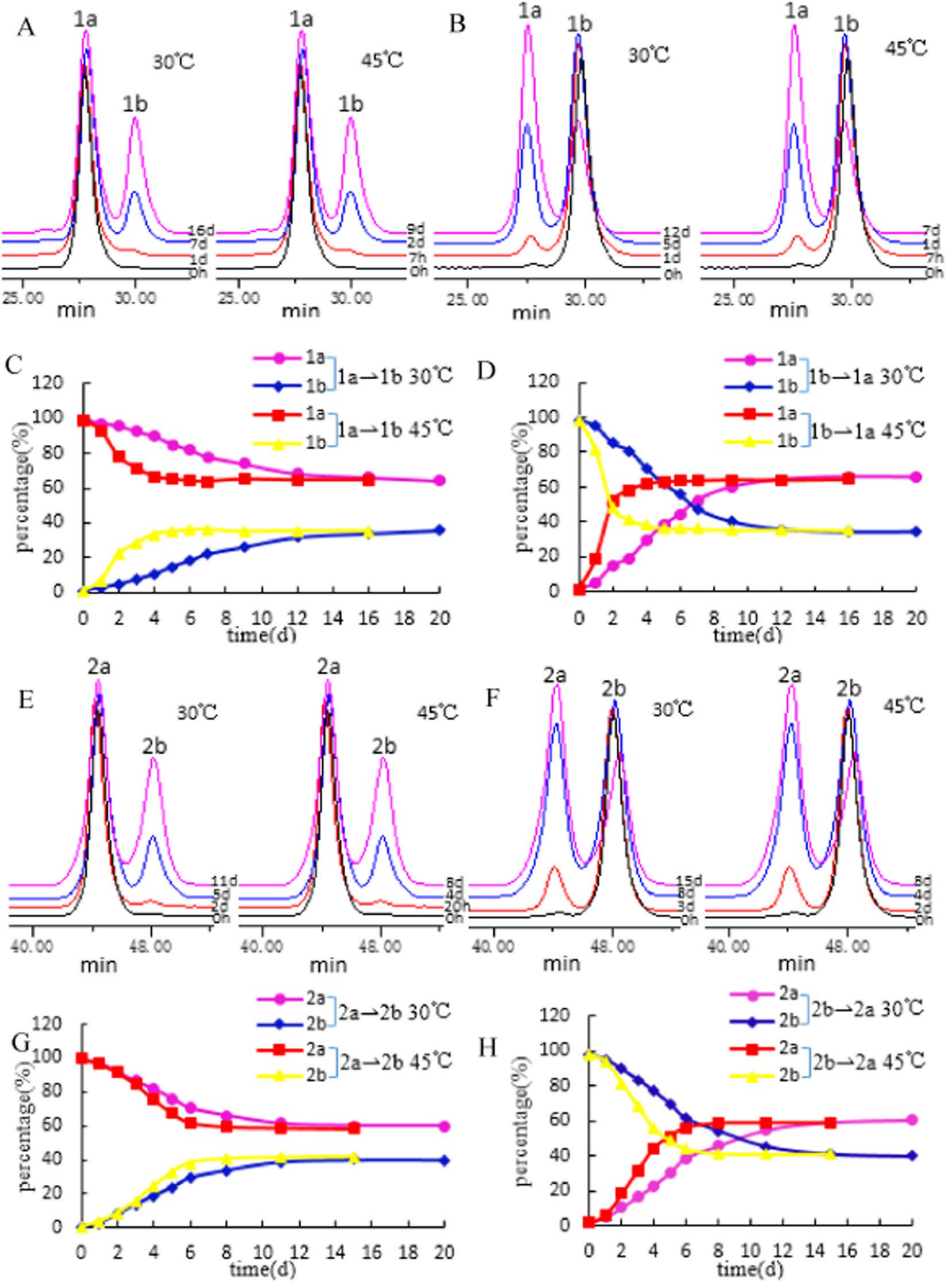
(**A**) The HPLC spectra of **1a** at different intervals in methanol; (**B**) The HPLC spectra of **1b** at different intervals in methanol; (**C**) The conversion curve of **1a** to **1b** in methanol; (**D**) The conversion curve of **1b** to 1a in methanol; (**E**) The HPLC spectra of **2a** at different intervals in methanol; (**F**) The HPLC spectra of **2b** at different intervals in methanol; (**G**) The conversion curve of **2a** to **2b** in methanol; (**H**) The conversion curve of **2b** to **2a** in methanol.

As shown in Fig. 6, for a **1a** sample solution maintained at 30 °C, the peak area (PA) of **1a** initially accounted for 97.19% of the total chromatographic PA; the PA started to decrease after 24 h, and a corresponding increase in the PA of **1b** was simultaneously detected. After 3 days, the PA of **1a** significantly decreased, while the PA of **1b** significantly increased. After 12 days, the reaction approached dynamic equilibrium, and the PA of **1a** accounted for 68.20% of the total PA. After 16 days, the reaction reached dynamic equilibrium, and the PA ratio of **1a** to **1b** was 2:1. For a **1a** solution incubated at 45 °C, the PA of **1a** started to decrease after 7 h, and a corresponding increase in the PA of **1b** was simultaneously detected. After 1 day, the PA of **1a** significantly decreased, while that of **1b** significantly increased. After 6 days, the reaction approached dynamic equilibrium, and the PA of **1b** accounted for 63.94% of the total PA. After 9 days, the reaction reached dynamic equilibrium, and the PA ratio of **1a** to **1b** was also 2:1.

For a **1b** sample solution stored at 30 °C, the PA of **1b** started to decrease after 7 h, and a corresponding increase in the PA of **1a** was simultaneously detected. After 2 days, the PA of **1b** significantly reduced and that of **1a** significantly increased. After 9 days, the reaction approached dynamic equilibrium. After 12 days, the reaction reached dynamic equilibrium, and the PA ratio of **1a** to **1b** was 2:1. For a **1b** sample solution incubated at 45 °C, the PA of **1b** started to decrease after 1 h, and a corresponding increase in the PA of **1a** was simultaneously detected. After 1 day, the PA of **1b** was significantly reduced, while that of **1a** significantly increased. After 5 days, the reaction approached dynamic equilibrium, and the PA of **1b** accounted for 59.79% of the total PA. After 7 days, the reaction reached dynamic equilibrium, and the PA ratio of **1a** to **1b** was also 2:1.

All results showed that **1a** was the preferential conformation in the pair of isomers that could interconvert, and the temperature affected both the stability and conversion speed of **1a** to **1b** and of **1b** to **1a** in a protic solvent, while their conversion rate was not affected. In addition, an interesting phenomenon was also found that under identical conditions, the conversion speed of **1b** to **1a** was faster than that of **1a** to **1b** in a protic solvent, and it increased with increasing temperature.

**2a** and **2b** were another pair of tautomers, of which **2a** was the preferred conformation. Similarly, the conversion speed from **2a** to **2b** was faster than that from **2b** to **2a**, and the conversion speed was faster at higher temperatures. As Fig. 3 and Fig. 4 show, under identical reaction conditions, the conversion speed to reach dynamic equilibrium for **1b⇌1a** was slower than that for **2b⇌2a**, which could be related to the spatial structure of the compounds (Fig. 6).

### Theoretical studies on tautomerism of the two pairs of tautomeric diterpenoids

To describe this conversion reaction mechanism, the transition state, bond length, and activation energy of the *two pairs* of tautomers were determined using density functional theory.

#### Transition state analysis of the conversion reaction

**Figure 7:**
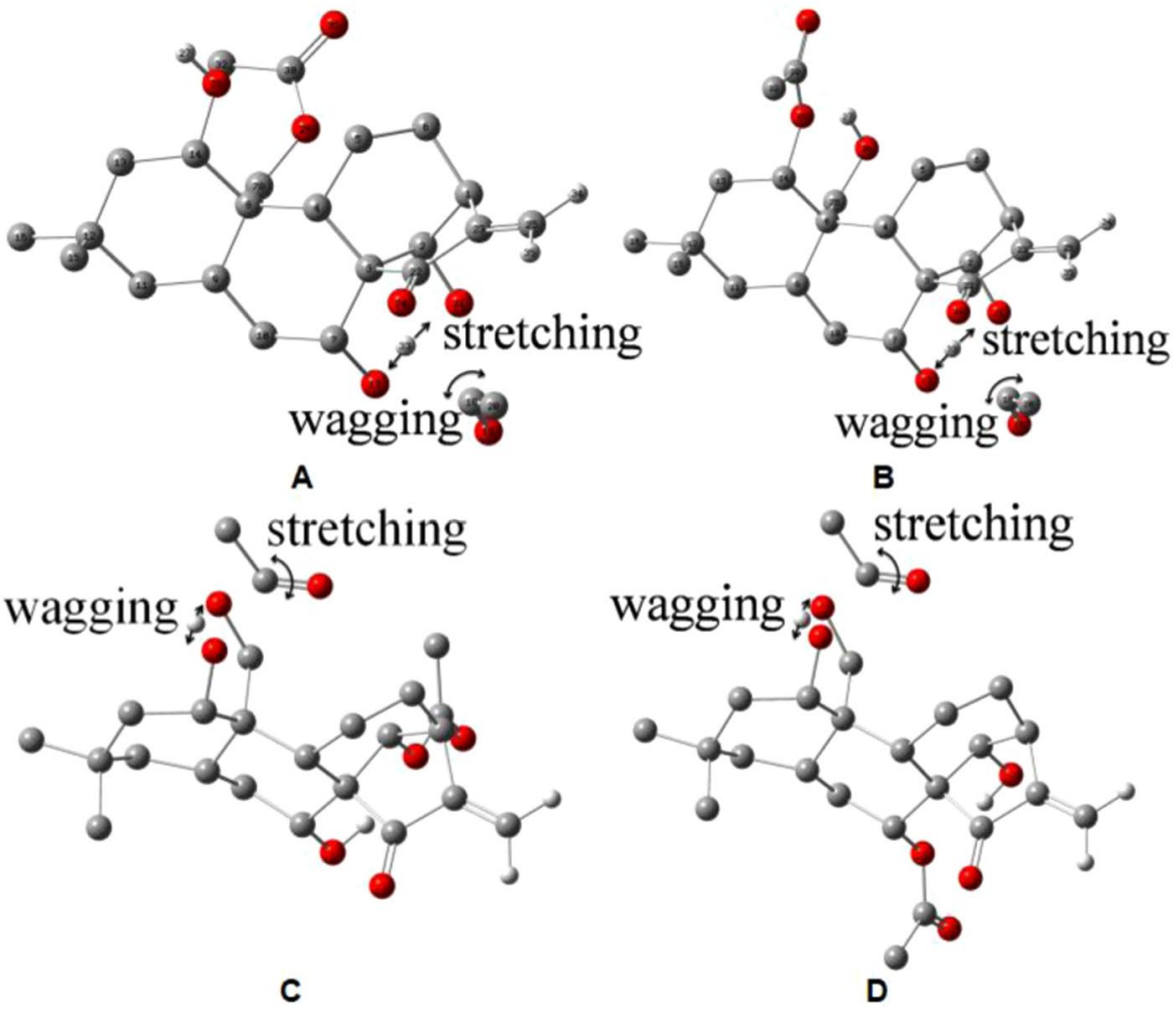
The vibration directions of virtual frequency of the three transition states TS1 (**A**), TS2 (**B**), TS3 (**C**) and TS4 (**D**). Note: Oxygen, carbon and hydrogen were colored red, gray and white, respectively. Hydrogen atoms on Saturated carbons are hidden for clarity.

Furthermore, the tautomerization mechanism of the two pairs of tautomeric diterpenoids was investigated, and the transition state calculations for the reactions were conducted using density functional theory. There is only a virtual frequency of −971.70 cm^−1^ for the transition state of the **1a⇌1b** reaction (TS1), which suggests that the optimal transitional state was obtained. The corresponding normal coordinates of the imaginary vibration modes for TS1 are shown in Fig. 7A. There was an obvious stretching vibration of H and a wagging vibration of the C-C-O bonds. The vibration frequency directions of TS1 point to **1a** and **1b**, respectively.

The minimum energy path (MEP) was also obtained through intrinsic reaction coordinate (IRC) calculations, which indicated that the transition state was correlated with both tautomers and that the transition state was located on the right reaction path.

Similarly, the transition state of the **2a⇌2b** reaction (TS2) was confirmed as well. There was only one virtual frequency of the transition state at −900.79 cm^−1^. A similar stretching vibration mode of H and C-C-O in TS2 could also be found (Fig. 7B).

#### Bond length analysis of the conversion reaction

**Figure 8:**
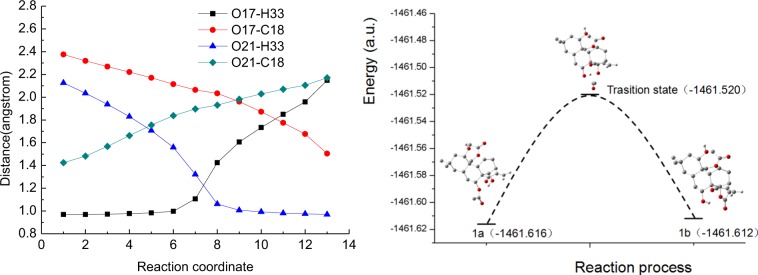
(**A**) The changing of four bond lengths of during the reaction process **1a⇌1b**; (**B**) The curve of energy change along the reaction pathway.

As shown in Fig. 8A, the tautomerization between compound **1a** and **1b** was conducted mainly by a proton transfer reaction according to the change in bond lengths. TS1 was formed at the seventh point of the reaction coordinate, and the bond lengths of O21-H33, O17-H33, O21-C18 and O17-C18 of TS1 were 0.112, 0.133, 0.192 and 0.205 nm, respectively. The distance between O21 and H33 decreased gradually before TS1 formation and then remained constant after TS1 formation, suggesting the formation of the O21-H33 bond. The distance between O17 and H33 remained constant before TS1 formation and increased gradually after TS1 formation, suggesting the cleavage of O17-H33. At the same time, the increasing distance between O17 and C18 suggested the cleavage of O17-C18, and the shortening distance between O21 and C18 suggested the formation of O21-C18. The results showed that the breaking and formation of H-O bonds and C-O bonds were the key factors causing tautomerism in compounds **1a** and **1b**.

#### Activation energy difference analysis of the conversion reaction

The curve of the energy change along the reaction pathway is shown in Fig. 8B. The energy changes of **1a⇌1b** also indicated that the conversion reaction had a low activation energy and that the conversion proceeded easily. The minimum energies for compounds **1a** and **1b** were −1461.612 a.u. and −1461.616 a.u., respectively. There was only a −0.004 a.u. (10.502 kcal·mol^−1^) energy difference between **1a** and **1b**. The tautomeric reaction of **1a** to **1b** was a forward reaction while that of **1b** to **1a** was the reverse reaction. The activation energies (Ea) of the forward reaction and reverse reaction were 252.0 kJ·mol^−1^ and 241.5 kJ·mol^−1^, respectively. The results indicated that the tautomeric reaction had low activation energies and that their conversion proceeded easily.

It is worth mentioning that there were also similar structural functional groups on the other pair of tautomeric diterpenoids. However, no new tautomerization reaction between -CH_2_-OH on site C1 and -OAc on site C20 between **1a** and **2a** and between **1b** and **2b** was found in the experiment. To explain this observation, the theoretical transition states of the **1a⇌2a** reaction (TS3) and **1b⇌2b** reaction (TS4) were calculated as well. The structures of TS3 and TS4 are shown in Fig. 7C,D. The activation energies of **1a⇌2a** and **1b⇌2b** were obviously higher than those of **1a⇌1b** and **2a⇌2b** (Table 3), indicating that the tautomeric reactions of **1a⇌2a** and **1b⇌2b** did not proceed as easily as those of **1a⇌1b** and **2a⇌2b**. Therefore, only the tautomerization reactions **1a⇌1b** and **2a⇌2b** were observed, while **1a⇌2a** and **1b⇌2b** were not.

**Table 3:** The activation energies of the reactions.

Reaction	Ea (kJ·mol^−1^)	Ea (kJ·mol^−1^)
	forward reaction	reverse reaction
1a → [TS1] → 1b	241.5	252.0
2a → [TS2] → 2b	244.0	252.9
1a → [TS3] → 2a	263.2	276.4
1b → [TS4] → 2b	253.5	267.5

#### Proton transfer process analysis of the conversion reaction

**Figure 9:**
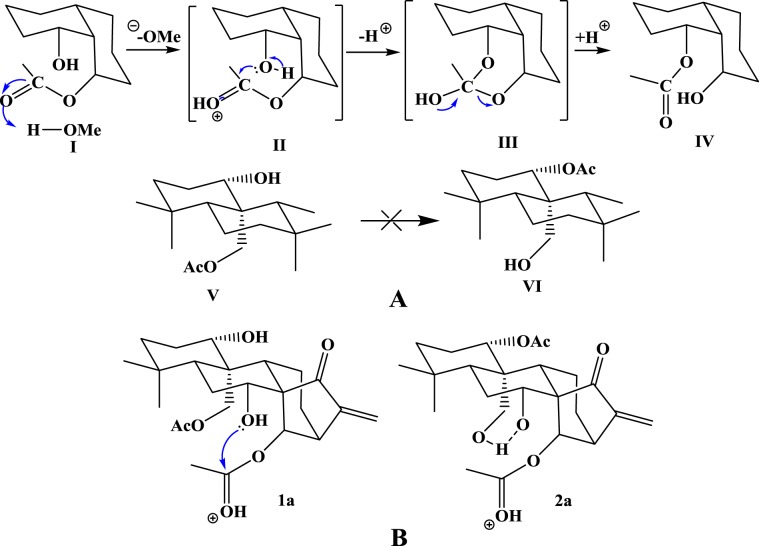
(**A**) The migration of acetate between C_14_ and C_7_, C_1_ and C_20_; (**B**) The migration of acetate between C_14_ and C_7_.

The tautomerism between both pairs of tautomeric diterpenoids was mainly through a proton transfer reaction. The migration of acetate between C_14_ and C_7_ is shown in Fig. 9A. The carbonyl group of **I** was first protonated. After the attack of oxygen from a hydroxy group in **II**, the transient orthoester **III** was obtained. Then, the collapse of orthoester **III** gave the final product **IV**. In contrast, the acetates on C_1_ and C_20_ were relatively unable to form orthoester intermediates due to their configuration exhibited in **V** and **VI**.

The speed of the **1b⇌1a** reaction is slower than that of the **2b⇌2a** reaction. **1a**, **1b**, **2a**, and **2b** were different from each other due to different substituents at C1, C20, C7 and C14. Compared with the hydroxyl group on C20 of **2a**, the acetate group on C_20_ of **1a** had one more chemical bond, so the acetate group with C_20_ on the axial bond of **1a** formed an intramolecular hydrogen bond with the hydroxy group on C_7_, which was not good for the transfer of the acetate group between C_7_ and C_14_. However, in molecule **2a**, the hydroxy group on C_20_ did not easily form hydrogen bonds due to the distance between the hydroxy groups on C_20_ and C_7_. Therefore, the transformation between **2a** and **2b** was much easier, as shown in Fig. 9B.

### Cytotoxicity assay

An MTT assay was performed to evaluate the cytotoxic effects of the tautomers against five human cancer cell lines, including HCT-116, A2780, NCI-H1650, BGC-823 and HepG2 (20170428, Beijing Bei Na Chuanglian Biotechnology Research Institute). The results are presented in Table 4.

**Table 4:** Cytotoxic activities of all tested compounds on five human cancer cell lines.

Sample	IC_50_ (μM)				
	HCT-116	HepG2	BGC-823	NCI-H1650	A2780
1	2.94 ± 0.06	3.07 ± 0.02	5.59 ± 0.19	>10	6.33 ± 0.34
2	2.45 ± 0.12	3.21 ± 0.09	4.17 ± 0.25	>10	5.61 ± 0.19
3	2.13 ± 0.81	2.20 ± 1.12	>10	5.68 ± 0.73	1.09 ± 0.13
4	1.77 ± 0.22	1.54 ± 0.32	1.31 ± 0.76	2.07 ± 0.36	1.42 ± 0.20
DDP	7.81 ± 0.14	>10	8.56 ± 1.05	>10	8.65 ± 0.59

DDP (cisplatin) was used as positive controls.

The MTT test showed that the cytotoxicities of the four compounds against the five human cancer cell lines were very different. In the future, extensive studies should be conducted to reveal the structure-activity relationship of cytotoxic diterpenoids for the discovery of affective antitumour drugs.

## Conclusions

In this paper, we isolated and identified four new 7,20-non-epoxy-*ent*-kaurane skeleton diterpenes as **1a**, **1b**, **2a** and **2b**. The interconversion experiments between **1a** and **1b** and between **2a** and **2b** in methanol solutions confirmed that they exists as two pairs of tautomers. Further analysis using density functional theory showed that the tautomeric reaction was closely related to the existence of a transition state, the change in bond length and the level of conversion energy. It is thought that proton transfer was the major tautomerization reaction mechanism between each pair of diterpene tautomers. In addition, the four diterpenes exhibited potent cytotoxicities against three or more tested human cancer cells. The results could serve as a valuable reference for the tautomerization mechanism of other tautomers and the study of the structure-activity relationship on cytotoxicities as anticancer drug precursors.

## Methods

### General experimental procedures

Optical rotation was measured using a SEPA-300 polarimeter (Horiba, Tokyo, Japan). NMR spectra were recorded on a Bruker Advance III spectrometer (Bruker, Billerica, Germany). Orientation separation and HRESIMS data were acquired using a UPLC-LTQ orbitrap (Thermo Fisher Scientific, Inc., Bremen, Germany). Semi-preparative HPLC was performed on a Waters 600/Waters 2487 (Waters, Milford, MA, USA) with a YMC (250 mm × 10 mm I.D. 5 μm) column. Column chromatography was performed either with silica gel (100–200 mesh and 200–300 mesh, Qingdao Marine Chemical Inc., Qingdao, China), MCI gel CHP 20 P (75–150 μm, Mitsubishi Chemical Corp., Tokyo, Japan), or with ODS (50 μm, YMC, Kyoto, Japan).

### Plant material

The aerial parts of *I. excisoides* were collected from Luanchuan County in Henan Province, China. The plant was authenticated by Xiao-Zheng Luo (Henan College of Traditional Chinese Medicine). A voucher specimen (No. 2011–0905) was deposited in the Laboratory of Research Center for Classic Chinese Medicines & Health Herbal Products.

### Extraction and isolation

The air-dried and powdered aerial parts of *I. excisoides* (10 kg) were extracted 3 times with water (320 L × 1.5 h) at 100 °C and concentrated to 0.1 g/mL. The concentrate was subjected to a D-101 macroporous resin column and successively eluted with EtOH-H_2_O (0%, 30%, 70% and 95%) to give four fractions (Fr. A-D). Fr. C (36 g, 70% EtOH elution) was purified with an MCI gel column (50 cm × 4 cm) and eluted with MeOH-H_2_O (3:7, 2 L; 5:5, 3 L; 7:2, 2 L; 10:0, 1 L) to yield four subfractions (Fr. C_1_-C_4_). Fr. C_2_ (16 g) was subjected to a silica gel column and eluted with petroleum ether-Me_2_CO (15:1, 10:1, 8:1, 5:1, 3:1, and 0:1) to afford four fractions (Fr. C_2a_-C_2d_). Two tautomeric mixtures of Fr. 1 (1.0 g) and Fr. 2 (0.7 g) were crystallized from Fr. C_2c_ using MeOH. **1a** (45 mg) and **1b** (30 mg) were isolated from the mixture of Fr. 1 (150 mg) by HPLC (acetonitrile-H_2_O, 29:71, 2.7 mL/min, peaks at 56.6 min and 61.2 min, respectively). The mixture of Fr. 2 (200 mg) was further purified by HPLC (acetonitrile-H_2_O, 31:69, 2.7 mL/min) to obtain **2a** (42 mg) and **2b** (31 mg) (71.2 min and 74.8 min, respectively).

#### 1α,7α-dihydroxy-14β,20-diacetoxy-ent-kaur-15-one (**1a**)

White powder; UV (MeOH) λmax (log ɛ): 235 (3.8); TLC (Sigel GF254 15 mm; petroleum ether /acetone 5:1, v/v) Rf: 0.42; *R*_t_ (RP-C_18_ HPLC, MeOH: MeCN 29:61, v/v): 27.46 min; infrared (IR) (cm^−1^): (stretching, ʋ; bending, *δ*; rocking, ρ): 3472 (br, O–H ʋ), 2934 (C–H ʋ), 1730 (C=O ʋ), 1649 (C=C ʋ), 1368 (C–H ρ), 1272 (C–O ʋ), 1159, 1043 (C–C ʋ), 968 (=C–H ʋ), 868 (C–H ʋ). ^1^H (proton) and ^13^C (carbon) data are listed in Table 1. High-resolution electrospray ionization mass spectrometry HR(ESI)MS: 457.21759 (calcd C_24_H_34_O_7_ Na^+^, *m/z* 457.21967).

#### 1α,14β-dihydroxy-7α,20-diacetoxy-ent-kaur-15-one (**1b**)

White powder; UV (MeOH) λmax (log ɛ): 235 (3.8); *R*_t_ (RP-C_18_ HPLC, MeOH: MeCN 29:61, v/v): 29.31; infrared (IR) (cm^−1^): (stretching, ʋ; bending, δ; rocking, ρ): 3445 (br, O–H ʋ), 2934 (C–H ʋ), 1731 (C=O ʋ), 1648 (C=C ʋ), 1368 (C–H ρ), 1242 (C–O ʋ), 1159, 1044 (C–C ʋ), 968 (=C–H ʋ), 866 (C–H ʋ). ^1^H (proton) and ^13^C (carbon) data are listed in Table 1. High-resolution electrospray ionization mass spectrometry HR(ESI)MS: 457.21744 (cal. C_24_H_34_O_7_ Na^+^, *m/z* 457.21967).

#### 1α,14β-diacetoxy-7α,20-dihydroxy-ent-kaur-16-en-15-one (**2a**)

White powder; UV (MeOH) λmax (log ɛ): 235 (3.3); *R*_t_ (RP-C_18_ HPLC, MeOH: MeCN 29:61, v/v): 44.93; infrared (IR) (cm^−1^): (stretching, ʋ; bending, δ; rocking, ρ): 3445 (br, O–H ʋ), 2935 (C–H ʋ), 1730 (C=O ʋ), 1648 (C=C ʋ), 1368 (C–H ρ), 1243 (C–O ʋ), 1158, 1044 (C–C ʋ), 968 (=C–H ʋ). ^1^H (proton) and ^13^C (carbon) data are listed in Table 1. High-resolution electrospray ionization mass spectrometry HR(ESI)MS: 457.21756 (cal. C_24_H_34_O_7_ Na^+^, *m/z* 457.21967).

#### 1α,7α-diacetoxy-14β,20-dihydroxy-ent-kaur-16-en-15-one (**2b**)

White powder; UV (MeOH) λmax (log ɛ): 235 (3.2); *R*_t_ (RP-C_18_ HPLC, MeOH: MeCN 29:61, v/v): 48.38; Infrared (IR) (cm^−1^): (stretching, ʋ; bending, δ; rocking, ρ): 3445 (br, O–H ʋ), 2934 (C–H ʋ), 1730 (C=O ʋ), 1648 (C=C ʋ), 1368 (C–H ρ), 1242 (C–O ʋ), 1158, 1044 (C–C ʋ), 968 (=C–H ʋ). ^1^H (proton) are ^13^C (carbon) data are listed in Table 1. High-resolution electrospray ionization mass spectrometry HR-(ESI)-MS: 457.21799 (cal. C_24_H_34_O_7_ Na^+^, *m/z* 457.21967).

### Tautomeric phenomenon and dynamic equilibrium between each pair of diterpene tautomers monitored by HPLC

Appropriate amounts of compounds **1a**, **1b**, **2a** and **2b** were separately weighed, and each compound was divided into two equal portions, placed in amber sample vials, and dissolved in methanol to obtain sample solutions with concentrations of 1.0 mg/mL. The solutions were sealed and separately stored at 30 °C and 45 °C. Each sample solution was analysed by HPLC every hour until tautomerization occurred. Then, the analysis was performed every 24 h until equilibrium was achieved. Chromatographic analysis was performed on a Waters 2996/Waters 2487 (Waters, Milford, MA, USA) equipped with a YMC C_18_ column (4.6 mm × 250 mm, 5 μm) with acetonitrile-water (23–70) implemented as the mobile phase for isocratic elution. The flow rate was 1.0 mL·min^−1^, and the column temperature was 25 °C. The detection wavelength was 230 nm. The stability of **1a**, **1b**, **2a**, **2b** and the time taken to reach equilibrium under different temperatures were observed.

### Theoretical studies on tautomerism of the two pairs of tautomeric diterpenoids

Furthermore, the tautomerization mechanism of the two pairs of tautomeric diterpenoids was investigated, and transition state calculations for the reactions were conducted using density functional theory. Gaussview 5.0 was used to build the molecular structures of the tautomers. The transition state was found at the B3LYP/6–31 + g (d) level and was confirmed by vibration frequency analysis. Then, the minimum energy path (MEP) was determined by using intrinsic reaction coordinate (IRC) calculations, and the imaginary vibrational mode of the transition state was studied. The stability of compounds **1a**, **1b, 2a**, and **2b** and their transition states were calculated by means of the density functional theory (DFT) method at the 6–31 + g(d) level. The calculations were performed using the Gaussian 09 software package.

### Cytotoxicity assay

Five human cancer cell lines (colon carcinoma cell line HCT-116, hepatic cancer cell line HepG2, ovarian cancer cell line A2780, lung cancer cell line NCI-H1650 and gastric cancer cell line BGC-823) (20170428, Beijing Bei Na Chuanglian Biotechnology Research Institute) were used for pharmacological experiments. All cells were cultured in RPMI-1640 medium supplemented with 10% foetal bovine serum in a humidified atmosphere with 5% CO_2_ at 37 °C. The cytotoxicity assay was performed according to the MTT method using 96-well microplates^12^.

In the test, each tumour cell was exposed to the test compound at concentrations of 1 × 10^−5^, 1 × 10^−6^, and 1 × 10^−7^ mol/L. The inhibitory rate of the cell growth was calculated according to the following formula: inhibition rate (%) = (OD _control_ − OD _treated_)/OD _control_ × 100. Finally, IC_50_ values were calculated using SPSS 16.0 statistical software.

## Supplementary information

Supplementary Information.
