# Author Correction: Isolation and identification of two pairs of cytotoxic diterpene tautomers and their tautomerization mechanisms

**DOI:** 10.1038/s41598-020-61343-1

**Published:** 2020-03-04

**Authors:** Li-Ping Dai, Xiao-Fei Li, Qing-Mei Feng, Ling-Xia Zhang, Qiu-Yan Liu, Er-Ping Xu, Hong Wu, Zhi-Min Wang

**Affiliations:** 1 School of Pharmacy, Henan University of Traditional Chinese Medicine, Zhengzhou, 450046 China; 2 Research Center for Classic Chinese Medicines & Health Herbal Products, Henan University of Traditional Chinese Medicine, Zhengzhou, 450046 China; 3 Institute of Chinese Materia Medica, China Academy of Chinese Medical Sciences, Beijing, 100700 China; 4 The Third Affiliated Hospital of Henan University of Traditional Chinese Medicine, Henan, China

Correction to: *Scientific Reports* 10.1038/s41598-020-58260-8, published online 29 January 2020

This Article contains errors in Table 4.

During the preparation of the manuscript the authors included wrong data in Table 4 that are from a different project. The correct Table 4 is included below as Table 1.

**Table 1:** Cytotoxic activities of all tested compounds on five human cancer cell lines.

Sample	IC_50_ (μM)				
	HCT-116	HepG2	BGC-823	NCI-H1650	A2780
1	4.90 ± 0.03	3.04 ± 0.02	7.78 ± 0.40	>10	2.10 ± 0.12
2	2.03 ± 0.02	2.82 ± 0.03	3.78 ± 0.30	>10	1.32 ± 0.21
3	1.20 ± 0.08	1.35 ± 0.02	4.81 ± 0.03	>10	2.78 ± 0.02
4	1.07 ± 0.03	1.21 ± 0.02	2.45 ± 0.06	>10	1.69 ± 0.04
DDP	8.23 ± 0.05	11.23 ± 0.01	23.43 ± 0.14	>10	2.00 ± 0.02

DDP (cisplatin) was used as positive controls.

