# Supplementary material for: Isolation and identification of two pairs of cytotoxic diterpene tautomers and their tautomerization mechanisms
Source: Sci Rep. 2020 Jan 29;10:1442. doi: 10.1038/s41598-020-58260-8 (PMC6989466; doi:10.1038/s41598-020-58260-8)
Supplement: Supplementary file 1 — Supplementary Information. [file 41598_2020_58260_MOESM1_ESM.doc]

Isolation and identification of two pairs of cytotoxic diterpene tautomers and their tautomerization mechanisms

Li-Ping Dai ^a, b #^, Xiao-Fei Li ^a #^, Qing-Mei Feng ^b^, Ling-Xia Zhang ^a^, Qiu-Yan Liu ^d^, Er-Ping Xu ^a^, Hong Wu ^a*^, Zhi-Min Wang ^b, c *^

^a^ School of Pharmacy, Henan University of Traditional Chinese Medicine, Zhengzhou, 450046, China; ^b^ Research Center for Classic Chinese Medicines & Health Herbal Products, Henan University of Traditional Chinese Medicine, Zhengzhou, 450046, China; ^c^ Institute of Chinese Materia Medica, China Academy of Chinese Medical Sciences, Beijing 100700, China. ^d^ The Third Affiliated Hospital of Henan University of Traditional Chinese Medicine.

**Electronic Supplementary Information**

The IR, HR-ESI-MS, NMR (1D and 2D) of compound 1a, 1b, 2a and 2b are available as supporting information. Please refer to Figures S1-40.

**Contents**

Figure S1. ^1^H-NMR spectrum of compound 1a and 1b( dynamic equilibrium for **1b****1a** )

Figure S2. ^13^C-NMR spectrum of compound 1a and 1b( dynamic equilibrium for **1b****1a** )

Figure S3. ^1^H-NMR spectrum of compound 2a and 2b( dynamic equilibrium for **2b****2a** )

Figure S4. ^13^C-NMR spectrum of compound 2a and 2b( dynamic equilibrium for **2b****2a** )

Figure S5. IR spectrum of compound 1a

Figure S6. HR-ESI-MS spectrum of compound 1a

Figure S7. ^1^H-NMR spectrum of compound 1a

Figure S8. ^13^C-NMR spectrum of compound 1a

Figure S9. DEPT spectrum of compound 1a

Figure S10. HSQC spectrum of compound 1a

Figure S11. ^1^H-^1^H COSY spectrum of compound 1a

Figure S12. HMBC spectrum of compound 1a

Figure S13. NOESY spectrum of compound 1a

Figure S14. IR spectrum of compound 1b

Figure S15. HR-ESI-MS spectrum of compound 1b

Figure S16. ^1^H-NMR spectrum of compound 1b

Figure S17. ^13^C-NMR spectrum of compound 1b

Figure S18. DEPT spectrum of compound 1b

Figure S19. HSQC spectrum of compound 1b

Figure S20. ^1^H-^1^H COSY spectrum of compound 1b

Figure S21. HMBC spectrum of compound 1b

Figure S22. NOESY spectrum of compound 1b

Figure S23. IR spectrum of compound 2a

Figure S24. HR-ESI-MS spectrum of compound 2a

Figure S25. ^1^H-NMR spectrum of compound 2a

Figure S26. ^13^C-NMR spectrum of compound 2a

Figure S27. DEPT spectrum of compound 2a

Figure S28. HSQC spectrum of compound 2a

Figure S29. ^1^H-^1^H COSY spectrum of compound 2a

Figure S30. HMBC spectrum of compound 2a

Figure S31. NOESY spectrum of compound 2a

Figure S32. IR spectrum of compound 2b

Figure S33. HR-ESI-MS spectrum of compound 2b

Figure S34. ^1^H-NMR spectrum of compound 2b

Figure S35. ^13^C-NMR spectrum of compound 2b

Figure S36. DEPT spectrum of compound 2b

Figure S37. HSQC spectrum of compound 2b

Figure S38. ^1^H-^1^H COSY spectrum of compound 2b

Figure S39. HMBC spectrum of compound 2b

Figure S40. NOESY spectrum of compound 2b


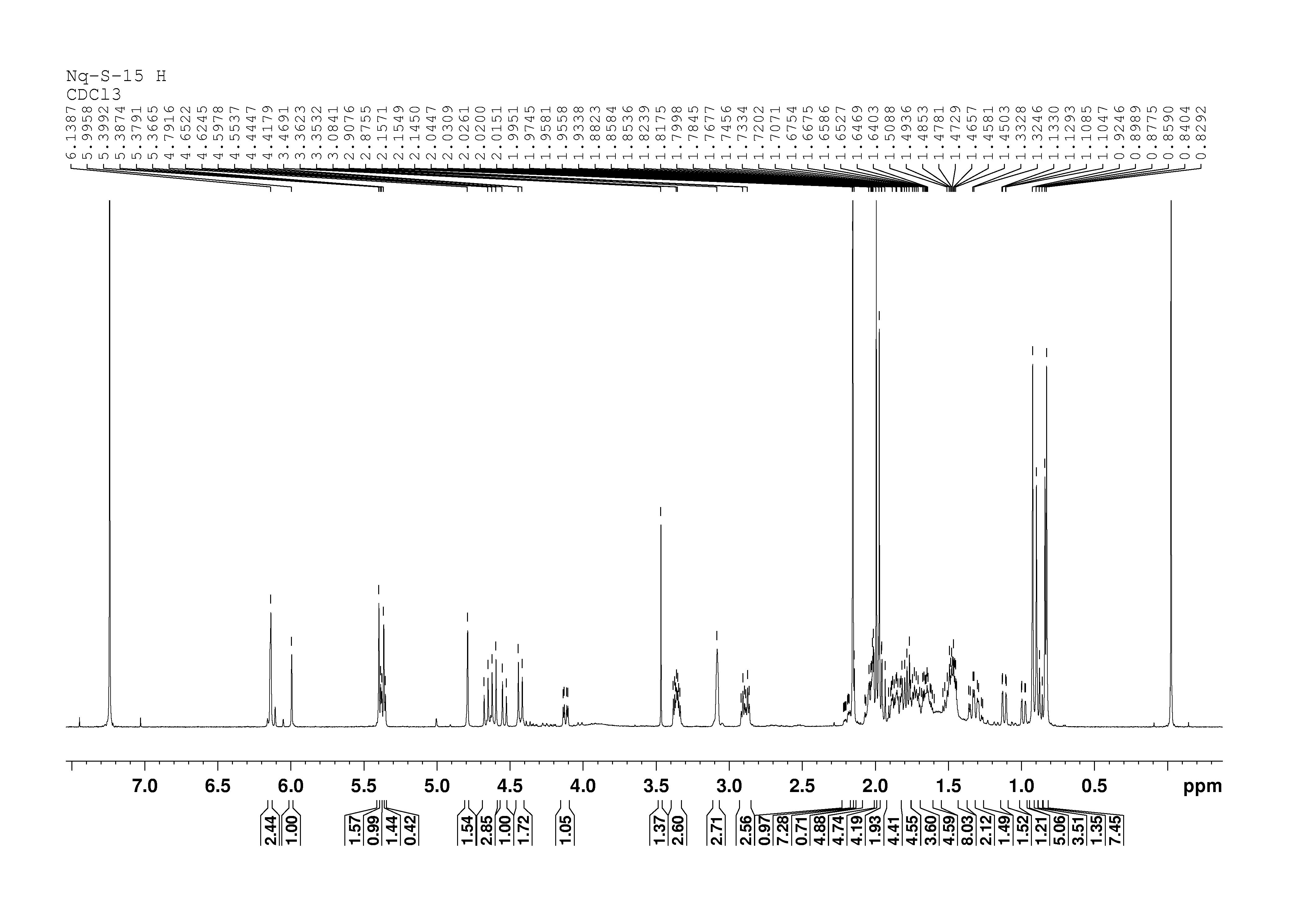


Figure S1. ^1^H-NMR spectrum of compound 1a and 1b


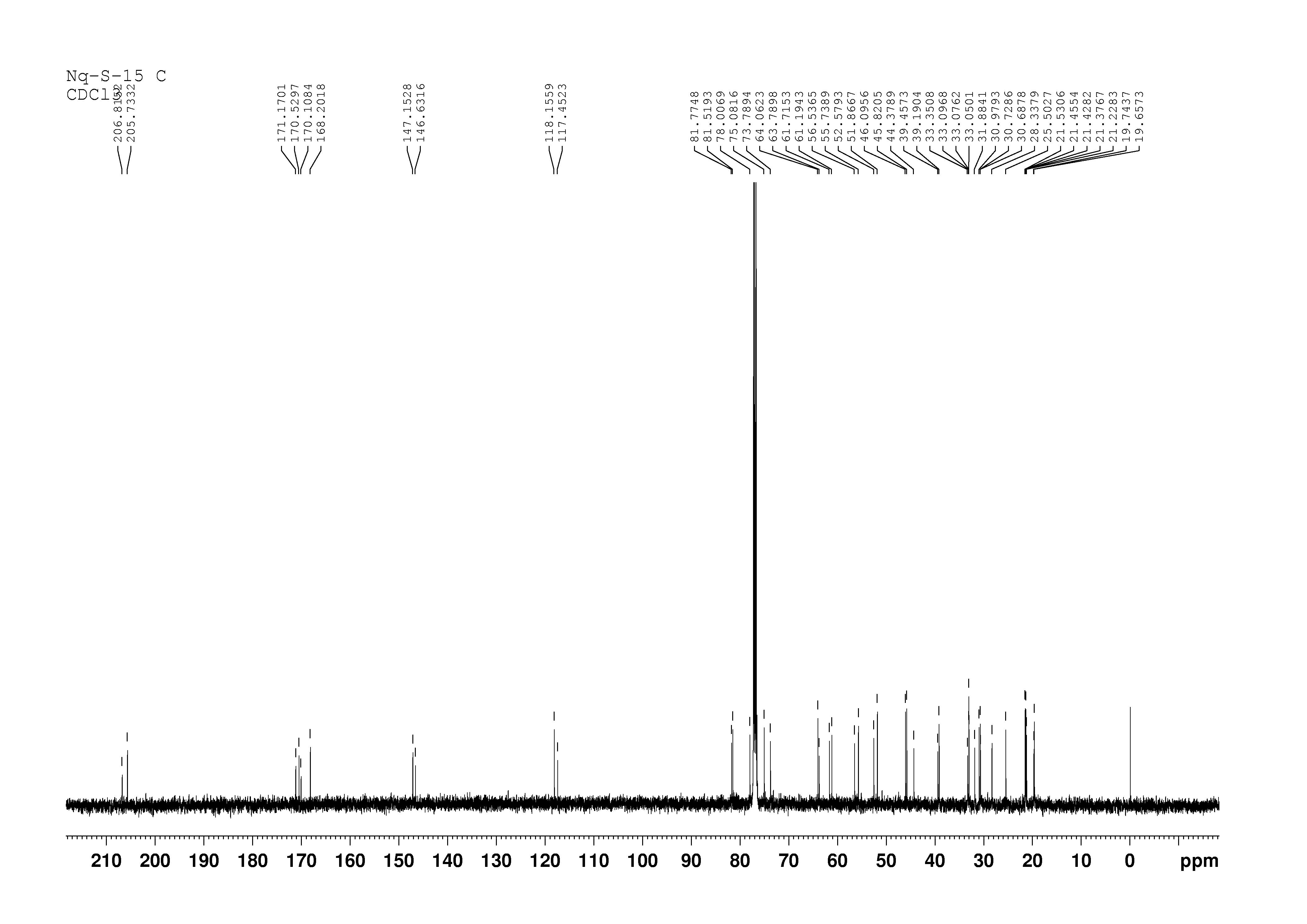


Figure S2. ^13^C-NMR spectrum of compound 1a and 1b

Figure S3. ^1^H-NMR spectrum of compound 2a and 2b

Figure S4. ^13^C-NMR spectrum of compound 2a and 2b


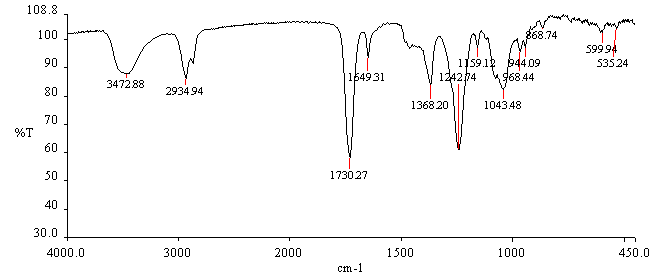


Figure S5. IR spectrum of compound 1a


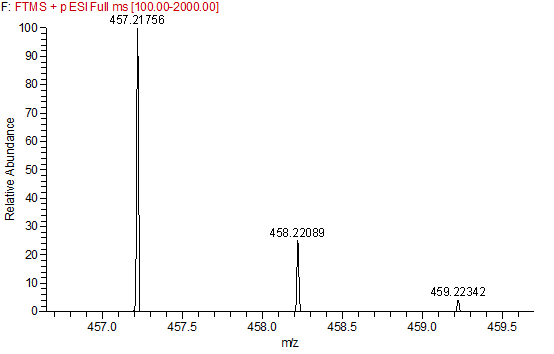


Figure S6. HR-ESI-MS spectrum of compound 1a

Figure S7. 1H-NMR spectrum of compound 1a

Figure S8. ^13^C-NMR spectrum of compound 1a

Figure S9. DEPT spectrum of compound 1a

Figure S10. HSQC spectrum of compound 1a

Figure S11. ^1^H-^1^H COSY spectrum of compound 1a

Figure S12. HMBC spectrum of compound 1a

Figure S13. NOESY spectrum of compound 1a


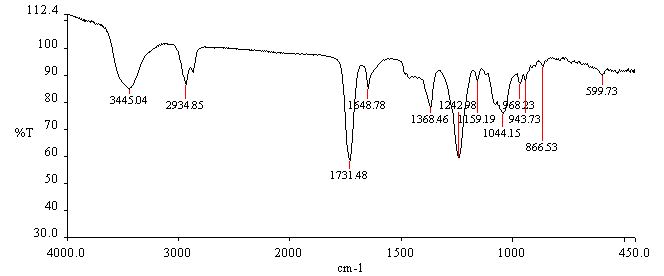


Figure S14. IR spectrum of compound 1b


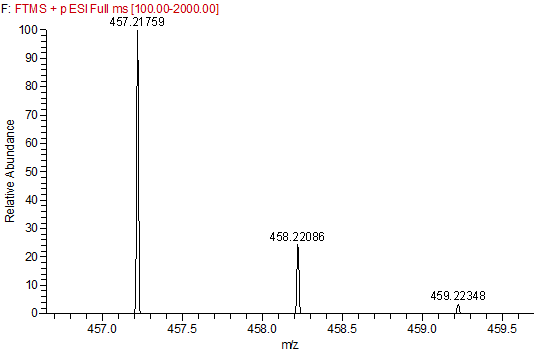
Figure S15. HR-ESI-MS spectrum of compound 1b

Figure S16. ^1^H-NMR spectrum of compound 1b

Figure S17. ^13^C-NMR spectrum of compound 1b

Figure S18. DEPT spectrum of compound 1b

Figure S19. HSQC spectrum of compound 1b

Figure S20. ^1^H-^1^H COSY spectrum of compound 1b

Figure S21. HMBC spectrum of compound 1b

Figure S22. NOESY spectrum of compound 1b


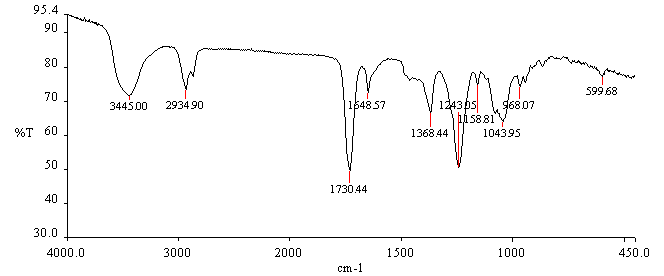


Figure S23. IR spectrum of compound 2a


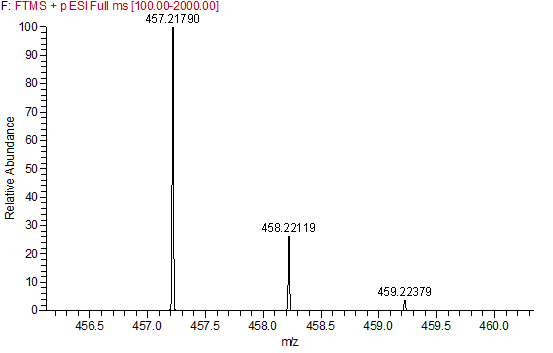


Figure S24. HR-ESI-MS spectrum of compound 2a

Figure S25. ^1^H-NMR spectrum of compound 2a

Figure S26. ^13^C-NMR spectrum of compound 2a

Figure S27. DEPT spectrum of compound 2a

Figure S28. HSQC spectrum of compound 2a

Figure S29. ^1^H-^1^H COSY spectrum of compound 2a

Figure S30. HMBC spectrum of compound 2a

Figure S31. NOESY spectrum of compound 2a


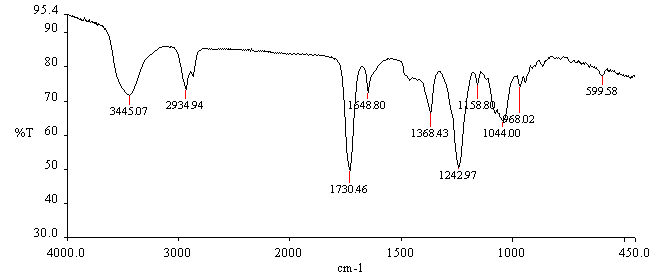


Figure S32. IR spectrum of compound 2b


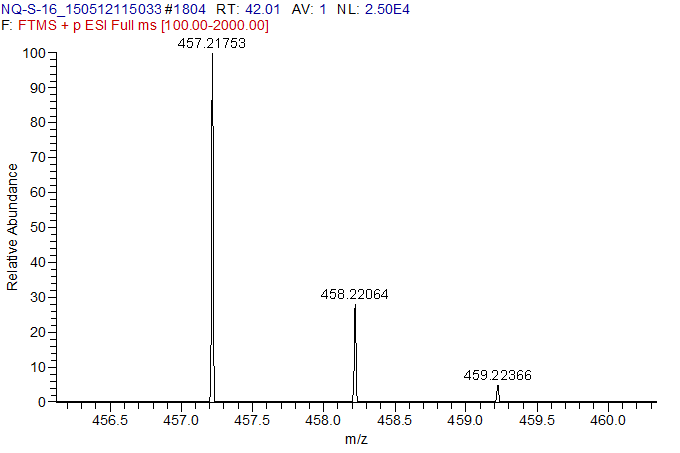


Figure S33. HR-ESI-MS spectrum of compound 2b

Figure S34. ^1^H-NMR spectrum of compound 2b

Figure S35. ^13^C-NMR spectrum of compound 2b

Figure S36. DEPT spectrum of compound 2b

Figure S37. HSQC spectrum of compound 2b

Figure S38. ^1^H-^1^H COSY spectrum of compound 2b

Figure S39. HMBC spectrum of compound 2b

Figure S40. NOESY spectrum of compound 2b
